# Alterations in bone mineral density and lower extremity lean mass after hip arthroscopy in a professional female Ironman triathlete: a case study

**DOI:** 10.1186/s40064-015-0859-0

**Published:** 2015-02-10

**Authors:** Sandro Manuel Mueller, Simone Braendli, Marco Toigo

**Affiliations:** Exercise Physiology Lab, Institute of Human Movement Sciences, ETH Zurich, Winterthurerstrasse 190, 8057 Zurich, Switzerland; Department of Orthopaedics, Laboratory for Muscle Plasticity, University of Zurich, Balgrist University Hospital, Forchstrasse 340, 8008 Zurich, Switzerland

**Keywords:** Dual-energy X-ray absorptiometry (DXA), Peripheral quantitative computed tomography (pQCT), Femoroacetabular impingement (FAI)

## Abstract

**Introduction:**

Femoroacetabular impingement is a pathomechanical condition of the hip, which is often treated through arthroscopic techniques. The ensuing immobilization period is associated with decreases in muscle mass and bone mass. To date, minimal knowledge is present about the development of tissue mass during the considerably short rehabilitation period before returning to competition in elite endurance athletes.

**Case description:**

Before and after surgery, a professional female Ironman triathlete underwent dual-energy X-ray absorptiometry and peripheral quantitative computed tomography measurements.

**Discussion and evaluation:**

Areal bone mineral density (aBMD) of the proximal femur and lower extremity lean mass decreased in the surgically treated lower extremity during the two-month period of immobilization after the hip arthroscopy. These losses were compensated for after only six weeks of rehabilitation. A similar progression of aBMD values was observed in the lumbar spine. The adaptational pattern in volumetric BMD (vBMD) and volumetric bone mineral content (vBMC) of the tibiae were more complex, but attained pre-immobilization values for most variables also after six weeks of rehabilitation. All other variables attained pre-immobilization values no later than nine months after the surgical intervention.

**Conclusions:**

The athlete showed a high plasticity of bone and lean tissue with an optimal short- and midterm outcome. Following a two months immobilization period after a hip arthroscopy, aBMD, vBMD and vBMC achieved pre-surgical levels after four months of rehabilitation in a female Ironman triathlete. A nine-month follow-up measurement confirmed the safety of the fast return to sport.

## Background

Femoroacetabular impingement (FAI) describes a pathomechanical condition of the hip (Ganz *et al*. 1991). It is characterized by an abnormal interaction between the acetabulum and the femoral head. It is categorized according to the position of the deformation. First, pincer impingements are characterized by a malformation of the acetabulum. Second, cam impingement is characterized by an altered morphology of the femoral head-neck junction. The third is a mixed type, where both characteristics of the pincer and cam impingements are present. The reasons for the development of most impingement lesions are unknown. Recently, it was proposed that pincer impingement results from metabolic or inflammatory diseases (Leunig *et al.*^2009^), and that cam impingement lesions are potentially caused by physical stress, *e.g.* excessive femoral loading (Lahner *et al.*^2014^), and genetics, *e.g*. the shape of the epiphyseal growth plate (Roels *et al.*^2014^).

Recently, the incidence of FAI in professional athletes of several different sports has been reported (Philippon *et al.*^2007^). The increased incidence in athletes gives rise to the assumption that chronic overload or traumata might be further responsible reasons for developing FAI. In athletes, favorable outcomes have been reported with arthroscopic treatment of patients with hip pain and osseous morphology consisted with FAI (Rath *et al.*^2012^). Hip arthroscopy for the treatment of FAI has demonstrated improved short- to midterm outcomes in athletes (Amenabar and O’Donnell 2013, McDonald *et al.*^2013^). On average, many professional athletes can resume their pre-injury training routines approximately four months after surgery (Malviya *et al.*^2013^). However, the treated lower extremity is immobilized after a hip arthroscopy for six to eight weeks (Stalzer *et al.*^2006^, Wahoff and Ryan 2011). This immobilization period inevitably leads to a decrease in muscle mass and possibly also to a reduction in bone mass since limb suspension has been shown to cause muscle atrophy after 4 weeks (Berg *et al.*^1991^) and bone loss after 24 days (Rittweger *et al.*^2006^) of suspension. Consequently, the goal of rehabilitation should not only be a fast return to pre-surgical levels of physical activity but also to counteract decreases in muscle mass and potentially bone mass. The mechanisms underlying the alterations in muscle mass, *i.e*. decrease during the immobilization period and increase during the rehabilitation period, might be positively influenced by (resistance) training and nutrition. In contrast, it is not known if the decrease in bone mass that occurs during the immobilization period can be fully recovered before athletes return to competition.

In the present case study, we investigated the acute alterations in bone mineral density (BMD), bone mineral content (BMC), and lean mass in a professional female Ironman athlete during the 9 months after hip arthroscopy as treatment for a FAI. Specifically, we were interested in the changes in bone values during the two months of lower extremity immobilization and the following rehabilitation period.

## Case description

### Participant

The participant was a 32-year old professional female Ironman triathlete. After a bicycle accident, the athlete developed progressive hip pain and was diagnosed with FAI with a labral tear. She elected to purse arthroscopic intervention. During the surgery, cystic changes at the anterior-inferior attachment of the labrum at the ligamentum transversum were resected. The whole labrum was degenerated, which necessitated a resection over the whole circumference. Femoral cartilage was intact and only superficial anteromedial cartilaginous fringes were observed, which were debrided. Acetabular cartilage delamination was observed between the 11 and 13 o’clock position and was treated by trimming of the acetabular rim between the 9 and 15 o’clock position. Due to the severity of the injury, the immobilization period after the surgical intervention persisted for four weeks, wherein the athlete was only allowed to walk on crutches. Starting from week 5 (25% weight bearing), partial weight bearing was progressively increased (*i.e*. 50% at week 6, 75% at week 7, 100% with crutches at week 8) to attain full weight bearing without crutches after week 8. In the two months after the surgery, physical activity was limited to 20 to 30 min of cycling per day. The 6 weeks thereafter (week 9 to week 14 post-surgery), cycling, swimming, and resistance training were allowed to be slowly increased to pre-surgery levels, while running exercise was limited to 15 to 30 min per day. Fourteen weeks after the surgery all restrictions were abrogated and the pre-surgery training routine could be resumed. Twenty-four weeks after the surgical intervention, the participant completed an Ironman. The participant was fully informed about the purposes, benefits and risks associated with the measurements and gave written informed consent to her participation in this study. The study was conducted in accordance with the Declaration of Helsinki. Additional informed consent was obtained from the participant for whom identifying information is included in this article.

### Experimental overview

**Figure 1:**
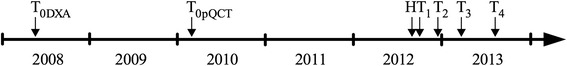
**Time line for the DXA and pQCT measurements during the study.** DXA, dual-energy X-ray absorptiometry; pQCT, peripheral quantitative computed tomography; H, hip arthroscopy; T_0_-T_4_, measurement points in time.

We had been following this athlete from 2008, which was at the point in time, where the participant started with triathlon training (T_0DXA_, Figure 1). Peripheral quantitative computed tomography (pQCT) was measured for the first time at the beginning of 2010, where the participant started her professional Ironman career (T_0pQCT_). One week after the hip arthroscopy, DXA and pQCT measurements were repeated (T_1_). Measurement point in time 2 (T_2_) corresponded to eight weeks of nearly complete immobilization. After six weeks of cycling, swimming, and resistance training, we performed pQCT and DXA measurements for the third time (T_3_). The last measurement point in time (T_4_) occurred five months after resuming the pre-surgery training routine, *i.e*. 9 months after the surgical intervention (Figure 1). For all testing, the participant arrived at the laboratory at the same time of day and the measurements were conducted in an identical order.

### Measurements

#### Dual-energy X-ray absorptiometry (DXA)

A densitometer (Lunar iDXA™, GE Healthcare, Madison, WI, USA) was used for the determination of body composition as well as areal BMD (aBMD) of the proximal femur and lumbar spine according to the manufacturer’s specifications. The delineation in regions of interest for the lower extremity was done automatically by the integrated software (enCORE, GE Healthcare, Madison, WI, USA; version 11.40.004). The leg regions of interest were defined as follows: upper boundary = horizontal line between femur and tibia; lower boundary = horizontal line through the tibio-talar joint; lateral boundaries = outer leg cuts.

#### Peripheral quantitative computed tomography (pQCT)

We measured volumetric BMC (vBMC) and volumetric BMD (vBMD) at 4, 14, 38, and 66% of tibia length in both legs with an XCT 3000 Scanner (Stratec, Pforzheim, Germany) as previously described (Anliker *et al.*^2011^).

### Data analysis

The statistical analysis for each parameter was performed based on the least significant change (LSC). A significant change was assumed if the change in a parameter was higher than LSC, whereby LSC is defined as 1.5 × typical error expressed as a coefficient of variation (CV; Hopkins 2000). For all DXA and pQCT variables, we used the CVs determined in our laboratory (Table 1).

**Table 1:** **Typical error expressed as a coefficient of variation (CV) for the DXA and pQCT variables**

**DXA proximal femur**	**CV left**	**CV right**	**DXA lumbar spine**	**CV**	**pQCT tibia**	**CV**
aBMD neck	1.2%	1.4%	aBMD L1	1.3%	vBMC_4%_	0.8%
aBMD upper neck	2.0%	1.9%	aBMD L2	1.6%	vBMD.tb_4%_	0.8%
aBMD lower neck	1.3%	1.5%	aBMD L3	1.7%	vBMD.tot_4%_	0.7%
aBMD shaft	0.7%	1.0%	aBMD L4	1.9%	vBMC_14%_	0.5%
aBMD trochanter	0.7%	1.1%	aBMD L1-L4	1.6%	vBMD.ct_14%_	0.3%
aBMD wards	2.0%	1.5%			vBMC_38%_	0.4%
aBMD total femur	0.5%	0.6%			vBMD.ct_38%_	0.2%
					vBMC.tib_66%_	0.2%
					vBMD.ct.tib_66%_	0.3%
**DXA lower extremity**	**CV left**	**CV right**				
Lower extremity lean mass	2.5%	2.4%				

DXA, dual-energy X-ray absorptiometry; pQCT, peripheral quantitative computed tomography; aBMD, areal bone mineral density; vBMC, volumetric bone mineral content; vBMD, volumetric bone mineral density; tb, trabecular; tot, total; ct, cortical; tib, tibia.

## Results

**Figure 2:**
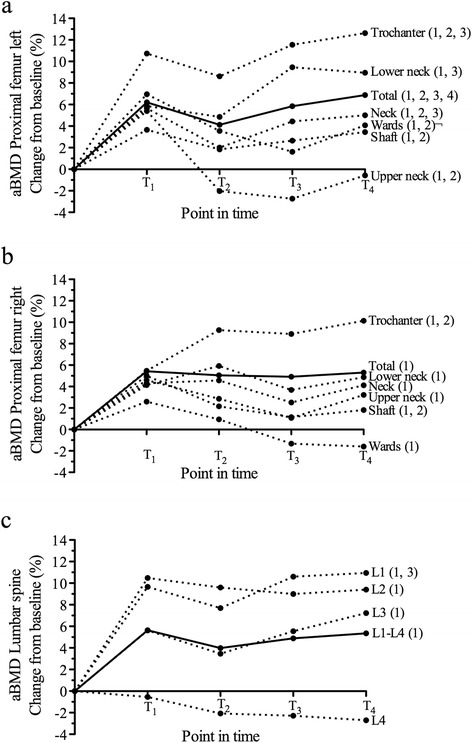
**Percentage change from baseline for areal bone mineral density (aBMD). a)** aBMD in the left proximal femur, **b)** aBMD in the right proximal femur, and **c)** aBMD of the lumbar spine at the four measurement points in time. Numbers in parenthesis represent increases/decreases higher than 1.5 × coefficient of variation for this variable in relation to the point in time before at the appropriate measurement site.

Total aBMD in the left proximal femur increased significantly from T_0DXA_ to T_1_ by 6.2%, from T_2_ to T_3_ by 1.7% and from T_3_ to T_4_ by 1.0%, while from T_1_ to T_2_, there was a significant decrease of 2.0% (Figure 2a). Decreases in aBMD from T_1_ to T_2_ were present at all distinct positions with the exception of the femur lower neck. The increase in aBMD from T_2_ to T_3_ was based on significant increases in aBMD at the neck, lower neck, and trochanter (Figure 2a). In the right proximal femur, total aBMD increased from T_0DXA_ to T_1_ by 5.4% and remained stable thereafter (Figure 2b). However, there was a significant increase in aBMD in the trochanter from T_1_ to T_2_ by 3.6%, while there was a significant decrease in aBMD in the shaft by 2.7% in the same period of time (Figure 2b). Total aBMD in the lumbar spine increased from T_0DXA_ to T_1_ by 5.6% and was slightly reduced from T_1_ to T_2_ by 1.6%. Total aBMD in the lumbar spine returned to pre-surgery values from T_2_ to T_4_. This progress was mainly due to alterations in L1 and L3, while L2 and L4 did not change from T_1_ to T_4_ (Figure 2c).

vBMC and vBMD in the left and right tibia increased at all measurement points in time from T_0pQCT_ to T_1_ (Table 2). In the left leg, only vBMC at the 38%-site decreased from T_1_ to T_2_. From T_2_ to T_3_, total vBMD at 4% of tibia length as well as vBMC at the 14% and 38%-site increased in the left leg, while cortical vBMD at the 66%-site decreased. All left leg values reached identical levels at T_4_ compared to T_1_. In the right leg, trabecular vBMD at the 4%-site and cortical vBMD at the 14%-, 38%- and 66%-site as well as vBMC at the 66%-site decreased from T_1_ to T_2_. These values increased from T_2_ to T_3_ with the exception of cortical vBMD at 38% of tibia length, which remained lower (Table 2).

**Table 2:** **Peripheral quantitative computed tomography (pQCT) vBMC and vBMD values for the left and right tibia**

	**Left leg**					**Right leg**				
	**T**_**0pQCT**_	**T**_**1**_	**T**_**2**_	**T**_**3**_	**T**_**4**_	**T**_**0pQCT**_	**T**_**1**_	**T**_**2**_	**T**_**3**_	**T**_**4**_
vBMC_4%_ (g · cm^−1^)	2.43	2.50+	2.51	2.48	2.48	2.46	2.55+	2.54	2.53	2.55
vBMD.tb_4%_ (mg · cm^−3^)	205	208+	207	207	207	202	208+	205−	207	208
vBMD.tot_4%_ (mg · cm^−3^)	263	266+	263	268+	266	247	257+	256	259+	258
vBMC_14%_ (g · cm^−1^)	1.81	1.86+	1.86	1.89+	1.87−	1.85	1.88+	1.88	1.88	1.88
vBMD.ct_14%_ (mg · cm^−3^)	1095	1113+	1116	1117	1113	1101	1116+	1110−	1119+	1113−
vBMC_38%_ (g · cm^−1^)	2.82	2.89+	2.86−	2.91+	2.91	2.80	2.82+	2.82	2.83	2.79−
vBMD.ct_38%_ (mg · cm^−3^)	1138	1150+	1148	1150	1148	1142	1153+	1147−	1142−	1145
vBMC.tib_66%_ (g · cm^−1^)	3.04	3.09+	3.11+	3.11	3.12+	3.09	3.16+	3.14−	3.16+	3.17+
vBMD.ct.tib_66%_ (mg · cm^−3^)	1094	1118+	1120	1114−	1119+	1097	1109+	1093−	1105+	1107

+, increase higher than 1.5 × coefficient of variation for this variable in relation to measurement point before; −, decrease higher than 1.5 × coefficient of variation for this variable in relation to measurement point before; vBMC, volumetric bone mineral content; vBMD, volumetric bone mineral density; ct, cortical; tb, trabecular; tib, tibia; tot, total.

**Figure 3:**
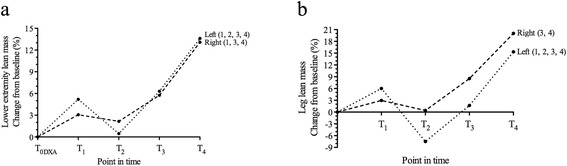
**Percentage change from baseline for lean mass. a)** Lower extremity lean mass and **b)** leg lean mass at the four measurement points in time. Numbers in parenthesis represent increase/decrease higher than 1.5 × coefficient of variation for this variable in relation to the point in time before at the appropriate measurement site.

Left lower extremity lean mass increased from T_0DXA_ to T_1_ (Figure 3a). During the immobilization period, lean mass in the left lower extremity was reduced by 4.5%, but it increased from T_2_ to T_3_ to the same extent. Furthermore, there was an increase in left lower extremity lean mass from T_3_ to T_4_ by 6.9%. In the right lower extremity, lean mass was increased from T_0DXA_ to T_4_ by 13.1%, but this increase was adjourned between T_1_ to T_2_ (Figure 3a). In the left leg, lean mass decreased by 12.7% from T_1_ to T_2_ but increased thereafter by 9.9% from T_2_ to T_3_ and by 13.4% from T_3_ to T_4_ (Figure 3b). Lean mass in the right leg was constant from T_0DXA_ to T_2_ and increased by 8.1% from T_2_ to T_3_ and by 10.6% from T_3_ to T_4_. The largest proportion of lower extremity lean mass loss between T_1_ to T_2_ was attributed to the legs (50 out of 62 g and 256 out of 304 g for the right and left lower extremity lean mass loss, respectively).

## Discussion and evaluation

In this case study of a professional female Ironman athlete, we found that aBMD of the proximal femur and lower extremity lean mass decreased in the surgically treated lower extremity during a two-month period of immobilization after hip arthroscopy. These losses were compensated for after only six weeks of rehabilitation. A similar recovery of aBMD values was present in the lumbar spine. The adaptational patterns in vBMD and vBMC of the tibiae were more complex. However, for most variables pre-immobilization values were attained after six weeks of rehabilitation, too. All other variables attained pre-immobilization values no later than nine months after the surgical intervention.

All bone parameters increased from the baseline measurement points in time to the measurement directly after the surgical intervention. In this four (DXA) and two (pQCT) year period, the participant increased training volume and intensity from a low to a professional level. After the rehabilitation and re-training period, the participant attained similar bone values as compared to before the surgery. These alterations in bone values can be explained by the different bone strains in the distinct periods of time. In theory, it is assumed that joint size adapts to maximum voluntary muscle force until the end of puberty, and, after growth plate closure, joint size cannot be further increased (Anliker *et al.*^2011^). As a consequence, the upper limit of maximum voluntary force is set by the given joint size after puberty, suggesting that maximum voluntary force has to be limited in order to prevent the system from damage (Anliker *et al.*^2011^, Anliker and Toigo 2012). Because of physical inactivity or immobilization maximum voluntary muscle force acting on bone is reduced, which in turn can lead to decreased bone mass and geometry (Rittweger and Felsenberg 2009). These decreases manifested themselves as lower aBMD in the lumbar spine and the left femur as well as reduced vBMD in the right tibia from T_1_ to T_2_. The observed reductions can be explained by the mechanostat theory (Frost 1987), which proposes that strain acting on the bone needs to exceed a certain lower threshold to allow (re)modelling of the bone (Frost 1990). With reloading through the still preserved joint area, maximum voluntary muscle force and thus bone strength thereafter can still recover. However, recovery would not lead to levels (significantly) above those achieved at the end of puberty (Anliker *et al.*^2011^). Cycling exercise during the immobilization period apparently was not an adequate stimulus for bone remodelling because cycling presumably does not lead to maximal bone strains, which arise typically from maximal muscle forces (Schiessl *et al.*^1998^). This result is supported by a previous study showing that vigorous cycling even leads to a reduction in aBMD at the femur neck and to a lower extent at the lumbar spine (Barry and Kohrt 2008). Furthermore, it was shown in a systematic review that professional road cyclists have a lower aBMD at the lumbar spine and femoral neck than age-matched controls (Olmedillas *et al.*^2012^).

In contrast to the alterations in the lumbar spine and the left proximal femur, total aBMD in the right proximal femur remained constant from T_1_ to T_4_. This indicates that total strain on the healthy lower extremity did not differ during the immobilization period. The similarity of total strain on the right lower extremity between before and during the immobilization period might be explained by two reasons. First, right lower extremity lean mass was maintained during the immobilization phase. Hence, the potential for maximal strains on the bone remained similar. Second, strain on the right lower extremity before the surgery was increased to reduce pain in the left lower extremity. This increased strain was maintained while walking on crutches or hopping without crutches leading to a high total strain on the right lower extremity. However, the alterations in aBMD at the different measurement positions (*e.g*. increase in aBMD at the trochanter and decrease at the wards from T_1_ to T_2_) point to the fact that total strain was similar to the strain before the surgical intervention but that the direction and size of the strain at the different measurement sites was different. These adaptations are explained by the goal of walking on crutches, which is to prevent an evasive movement. Consequently, it can be assumed that the direction of the acting forces and strains were different after the surgical intervention relative to pre-surgery, where evasive and compensatory movements had been present. This motion pattern/loading situation is supported by a study showing that the involved and non-involved lower extremities are differentially loaded during three-point gait crutch walking and that these loads are different from normal walking (Li *et al.*^2001^).

The alterations in bone mass and density in the tibiae were different compared to the changes in the proximal femura and lumbar spine. In the right tibia, vBMC and/or vBMD decreased at all measurement positions from T_1_ to T_2_, while in the left tibia decreases were only present at the 38%- and 66%-site. The more pronounced reduction in vBMD and partly vBMC in the right tibia might be explained by the activity and strain before the immobilization period. Due to the pain in the left hip and left lower extremity, load during everyday activity and training was distributed unevenly between both legs, resulting in a higher load on the right leg. As a consequence, stress was higher in the right tibia during everyday activity and training. Support for this disproportional load distribution comes from the observed side-to-side difference in leg lean mass (data not shown). This side-to-side asymmetry resulted in a higher modelling/remodelling threshold in the right tibia. The relief of a large portion of this stress during the immobilization period shifted the strain on the right tibia permanently below the remodelling threshold, which resulted in a decrease in net balance of bone. Since the strain on the left tibia was already lower before the immobilization period, the remodelling threshold was lower. Hence, the reduction in bone strain due to the immobilization period did not lead inevitably to a negative net balance of bone mass.

The increases in aBMD in the lumbar spine and the left proximal femur as well as the increases in vBMD in both tibiae after T_2_ can be explained by the return to everyday activity and by the intensified resistance training during this period. It is well known that resistance training involves high strains on the bone leading potentially to an increase in aBMD (Kelley *et al.*^2001^). The effectiveness of the resistance training in our participant is visible by the massive increase in lower extremity lean mass from T_2_ to T_4_. The adaptations of vBMD and vBMC of the tibiae after the intensified training were returns to pre-immobilization levels. However, there were site-specific alterations within both tibiae. The non-linearity of these results might be explained by at least two factors. First, there is a high variability of vBMD and vBMC adaptations along the tibia to immobilization (Rittweger *et al.*^2005^). Second, bone turnover is not bound to the site of the bone (Parfitt 2002).

Our result that aBMD, vBMD and vBMC recovered completely after an immobilization period is in line with a bed rest study of Rittweger and Felsenberg (2009), which stated that re-accrual rate of vBMC in the tibia after an immobilization period is very high. Furthermore, they showed that recovery of bone followed neuromuscular recovery (Rittweger and Felsenberg 2009), which is in line with our results of lower extremity lean mass increase. Remarkably, the recovery of aBMD in the lumbar spine and proximal femur took only six weeks in the professional athlete. This is considerably shorter than the recovery time of aBMD after two months of complete immobilization after hip surgery in inactive women (12–18 months; Ito *et al.*^1999^) and the recovery time of aBMD in the hip and lower limb after lower-limb fractures in adolescents (>6 months; Ceroni *et al.*^2013^).

The measurement point in time T_1_ was one week after the surgical intervention. It might be possible that aBMD, vBMD, vBMC and muscle mass already decreased in the time frame between surgery and T_1_. After inspection of the individual data, it can be assumed that a decrease in muscle mass might have taken place, because muscle mass was markedly higher at T_4_ compared to T_1_. In contrast, this progression was not present for aBMD, vBMD and vBMC. Therefore, we assume that no significant decrease in aBMD, vBMD and vBMC took place between the surgical intervention and T_1_.

## Conclusions

A two-month immobilization period after hip arthroscopy led to marked decreases in aBMD in the proximal femur of the lower extremity that underwent surgery as well as the lumbar spine and also to a loss of lean mass in the affected lower extremity. These decreases were reversed after only six weeks of resistance, cycling, and swim training with a negligible amount of running exercise. Consequently, the athlete showed a high plasticity of bone and lean tissue with an optimal short- and midterm outcome. A return to pre-surgery levels for aBMD, vBMD and vBMC in a professional Ironman triathlete is possible after only 4 months of rehabilitation after hip arthroscopy. A nine-month follow-up measurement confirmed that the fast return to sport had no negative impact on bone variables and was therefore safe. This case study indicated that a hip arthroscopy for treatment of FAI is a promising and safe option to allow a fast and symptom-free return to sport in a professional athlete. Nonetheless, further studies are needed to shed light on the progression of bone and muscle variables in different sports disciplines with distinct loading conditions.
